# Depression profile in malignancy patients attending otorhinolaryngology clinic

**DOI:** 10.1007/s00405-020-06289-w

**Published:** 2020-08-17

**Authors:** Meera Niranjan Khadilkar, K. Keshava Pai, Thripthi Rai, Vijendra Shenoy, Deviprasad Dosemane, Sushmitha Kabekkodu

**Affiliations:** 1 Department of Otorhinolaryngology and Head and Neck Surgery, Kasturba Medical College, Mangalore, Manipal Academy of Higher Education, Manipal, Karnataka, Mangalore, 575001 India; 2 Department of Psychiatry, Kasturba Medical College, Mangalore, Manipal Academy of Higher Education, Manipal, Karnataka, Mangalore, 575001 India

**Keywords:** Malignancy, Otorhinolaryngology, PHQ-9, Depression

## Abstract

**Purpose:**

Patients with malignancy quite often suffer from physical as well as psychological symptoms due to the shattering diagnosis, and prolonged, incapacitating management. The frequency of the depressive disorder in malignancy is around 8–40%. The present study aims at analysing the socio-demographic profile and magnitude of depressive disorders in patients with malignancy.

**Methods:**

A cross-sectional study was conducted in malignancy patients attending an Ear Nose Throat department using the PHQ-9 questionnaire.

**Results:**

Total PHQ-9 score ranged from 0 to 19; the mean score was 8.46. Major depressive disorder was seen in 4 (8%) cases, while other depressive disorder occurred in 22 (44%) cases. Mild severity of symptoms was noted in 15 (30%) of the patients. High statistical significance was noted between PHQ-9 score for MDD and other depressive disorder (*p* value < 0.001).

**Conclusion:**

The profile of depressive disorders in malignancy varies; PHQ-9 can be used as a good tool for early detection.

**Electronic supplementary material:**

The online version of this article (10.1007/s00405-020-06289-w) contains supplementary material, which is available to authorized users.

## Introduction

Patients with malignancy quite often suffer from physical as well as psychological symptoms due to the shattering diagnosis, and prolonged, incapacitating management. Around 32% such cases suffer from some form of psychological stress. Furthermore, the frequency of the depressive disorder in malignancy is around 8–40% [1, 2]. The PHQ-9 questionnaire was created for regular screening, diagnosis and measurement of symptoms of depression in centers treating patients with cancer [3, 4]. This tool has demonstrated good specificity and sensitivity for diagnosis, particularly in chronic disorders [1]. Studies suggest that head and neck malignancy patients diagnosed with the major depressive disorder had 25% higher mortality when compared to those without depression, independent of TNM classification [5, 6]. The present study aims at analysing the socio-demographic profile and magnitude of depressive disorders in patients with malignancy, and the correlation with PHQ-9 score.

## Materials and methods

We conducted a cross-sectional study over six months, on 50 patients who visited Ear Nose Throat (ENT) department with ENT or non-ENT related malignancies. Patients below 18 years of age and all patients with a known history of depressive disorder were excluded. Written informed consent was acquired from the participating subjects. Social as well as demographic data and details about malignancy were noted from medical records. Patients were then requested to complete the PHQ-9 questionnaire to screen for depressive disorders, by marking nine Diagnostic and Statistical Manual of Mental Disorders, 4th Edition (DSM-IV) criteria of major depressive disorder on a scale of zero (not at all) to three (nearly every day) in the prior fortnight. PHQ-9 sum scores were used for analysis. A score higher than 10 is positive for depression. A diagnosis of Major depressive disorder (MDD) was established if five symptoms or more were seen for at least more than half the days in the prior fortnight, one of them being anhedonia or low mood. A diagnosis of other depressive disorder was established if 2–4 symptoms of depression were seen for at least more than half the days in the prior fortnight, one of them being low mood or anhedonia [3]. If found to have depression, they were referred to the department of Psychiatry for further management. Statistics was analysed with SPSS 25 software. A *p*-value below 0.05 was considered statistically significant. Institutional ethical committee clearance was obtained.

## Results

Age range was from 39 to 90 years; mean age was 57.26 years. Male: female ratio was 3.54. The most common occupation was manual labour (58%), followed by agriculture (16%). (Table 1) No statistical significance was noted on comparison of PHQ-9 score with age, gender and occupation (*p* value 0.125, 0.253 and 0.096 respectively). Dysphagia was the most frequent presenting ENT symptom in 15 (30%) patients, followed by ulcer in the mouth in 12 (24%) patients. Duration of ENT symptoms ranged from 1 month to 2 years, with a mean duration of 4.12 months. Oral cavity was the commonest site of malignancy in 22 (44%) patients, followed by 8 (16%) patients with malignancy hypopharynx. Seventeen (34%) patients presented with stage III malignancy, followed by 13 (26%) patients with stage IVA malignancy. Total PHQ-9 score ranged from 0 to 19; mean score was 8.46. Major depressive disorder was seen in 4 (8%) cases, while other depressive disorder occurred in 22 (44%) cases. Mild severity of symptoms was noted in 15 (30%) of the patients. High statistical significance was noted between PHQ-9 score for MDD and other depressive disorder (*p* value < 0.001). (Table 2).

**Table 1:** Table showing socio-demographic profile of patients

Variables	Number (%)
Age	
< 30 years	0 (0%)
31–40 years	4 (8%)
41–50 years	6 (12%)
51–60 years	21 (42%)
> 60 years	19 (38%)
Sex	
Male	39 (78%)
Female	11 (22%)
Occupation	
Farmer	8 (16%)
Housewife	5 (10%)
Labourer	29 (58%)
Office: below clerk	4 (8%)
Self-employed	2 (4%)
Unemployed	2 (4%)
Presenting symptoms	
Swelling inside mouth	9 (18%)
Mouth ulcer	12 (24%)
Neck swelling	11 (22%)
Dysphagia	15 (30%)
Dyspnoea	4 (8%)
Voice change	5 (10%)
Otalgia	2 (4%)
Ear block	1 (2%)
Nasal obstruction	3 (6%)
Epistaxis	1 (2%)
Co-morbidities	
Diabetes mellitus	2 (4%)
Hypertension	7 (14%)
Ischemic heart disease	2 (4%)
Retroviral disease	2 (4%)
Post-operative status	1 (2%)
Post radiotherapy	7 (14%)
Post chemotherapy	1 (2%)
Psoriasis	1 (2%)
Hepatitis C Virus infection	1 (2%)
Second malignancy	1 (2%)
Habits	
Tobacco chewing	26 (52%)
Smoking	18 (36%)
Alcohol intake	19 (38%)
Diagnosis	
Carcinoma oral cavity	22 (44%)
Carcinoma oropharynx	5 (10%)
Carcinoma hypopharynx	8 (16%)
Sinonasal malignancy	2 (4%)
Thyroid malignancy	1 (2%)
Carcinoma supraglottis	5 (10%)
Carcinoma glottis	1 (2%)
Carcinoma subglottis	1 (2%)
Carcinoma lung	2 (4%)
Carcinoma breast	1 (2%)
Carcinoma parotid	1 (2%)
Hodgkin’s lymphoma	1 (2%)
TNM staging	
I	1 (2%)
II	11 (22%)
III	17 (34%)
IV A	13 (26%)
IV B	7 (14%)
IV C	1 (2%)
Diagnosis based on PHQ-9	
Control	24 (48%)
Major depressive disorder	4 (8%)
Other depressive disorder	22 (44%)
Severity of depressive symptoms	
Nil	1 (2%)
Minimal	12 (24%)
Mild	15 (30%)
Moderate	14 (28%)
Moderately severe	8 (16%)

**Table 2:** Table showing statistical correlation

	*p* value
Mann–Whitney test	
Tobacco	0.28
Smoking	0.808
Alcohol	0.055
Post-operative status	0.466
Post chemotherapy	0.101
Post radiotherapy	0.152
Spearman test of linear correlation	
Duration of symptoms	0.259
Independent *t* test	
MDD vs other depressive disorder	< 0.001
Control vs MDD	< 0.001
Control vs other depressive disorder	< 0.001
Pearson's Chi-square test of association	
Occupation	0.096
Sex	0.253
Staging vs PHQ-9 score	0.82
Staging and PHQ-9 diagnosis	0.677
ENT diagnosis vs PHQ-9 diagnosis	0.286
Pearson's test of correlation	
Age and PHQ-9 score	0.125

## Discussion

Around 32% of malignancy patients have at least one mental disorder, whereas affective disorders are frequently seen as psychological comorbidities. Furthermore, the frequency of depression in malignancy patients is projected to be 8–25%, increasing to 30% in palliative care settings [1]. Hartung et al. concluded that approximately 50% of cancer patients would require referral for additional diagnostics to detect patients with actual MDD [7]. Nevertheless, depressive disorders often remain undiagnosed and untreated in malignancy patients [8]. In the present study, the prevalence of other depressive disorder and MDD was found to be 44% and 8% respectively.

The PHQ-9 questionnaire contains nine questions, each centering on the DSM-IV criteria to diagnose MDD and matches with the latest DSM-5 criteria. Sleep disturbance, change in appetite, loss of energy, and psychomotor agitation are the somatic symptoms, while depressed mood, loss of interest, reduced concentration, suicidal thoughts and worthlessness are the psychological symptoms [1].

Even though there was a male preponderance in the present study, severity of depressive symptoms was slightly higher in women, and so was the incidence of major depressive disorder, than in men. The findings are similar to that by Fan et al., which showed that women have a greater possibility of developing depression as a result of head and neck malignancy. It could possibly be due to lack of social support, the necessity to undergo disfiguring surgery, hormonal changes [5].

Fan et al. concluded that depression was more frequent in the younger population having head and neck malignancy, which could be due to the disease impact and the treatment being more intrusive upon lifestyle [5]. On the contrary, Saracino et al. concluded the frequency of substantial symptoms of depression in the older population ranged from 8–16%, which could be up to 23% while assessing mild symptoms of depression [9]. In the present study, the majority of the cases were in the age group of 50–70. Severity of depressive symptoms was higher in this age group, and so was the prevalence of depressive disorders; other depressive disorder was commoner in this group than MDD.

Depression in the elderly population is frequently underestimated and under-treated, possibly because the symptoms are reported differently. In malignancy, diagnosis of depression can be difficult, which may be due to the similarity between the diagnostic criteria for depression, and the symptoms related to cancer and/or treatment, like lethargy, disturbances in sleep, decreased interest in sexual activity [9].

In the present study, manual labour was the predominant occupation (58%). MDD was seen in 8%, other depressive disorder in 44%, while the large majority (48%) were not found to have a depressive disorder. Interestingly, other depressive disorder was found to be most frequent in manual labourers and farmers. However, the severity of depressive symptoms was highest in housewives and unemployed persons (PHQ-9 score of 9), followed by labourers and farmers (mean PHQ-9 score of 8.9 and 8.25 respectively). Fan et al. found a higher chance of developing depression in unemployed, retired and subjects having low-income when compared to patients with higher income and white-collar jobs. Financial constraints and family problems may cause additional distress [5].

Even though the commonest malignancy in the study group was oral cancer (44%), followed by hypopharynx (16%), the severity of depressive symptoms was found to be maximum in paranasal sinus malignancy (score of 17), followed by parotid malignancy and lymphoma (score of 16 each). However, other depressive disorders and MDD were more frequent in oral cancer when compared to other malignancies. Fan et al. found the maximal risk of depressive disorders in hypopharyngeal and oropharyngeal cancer [5].

Grapp et al. concluded that the somatic symptoms of PHQ-9 are beneficial in the identification of at-risk malignancy cases having mild/moderate depressive disorder, thus preventing underestimating depression in malignancy. As patients tend to present with somatic, rather than psychological complaints, clinicians must be in a position to recognize as well as manage them, and identify related depressive disorders [1]. Nikendei et al. found that cancer patients showed more somatic symptoms of depression when compared to patients without cancer, thus improving clinical awareness [8].

In the present study, the majority of the patients (78%) presented within 6 months of developing otorhinolaryngological symptoms, among whom, other depressive disorder was predominant. No statistical significance was noted between duration of symptoms and PHQ-9 score (*p* value 0.259). Thirty-four percent cases presented in stage III of malignancy. Nevertheless, there was not much of variation in the severity of depression and incidence of other depressive disorder and MDD between stage II and stage IV (score of 7.23–9.73). No statistical significance was noted between malignancy stage and PHQ-9 score or depressive disorder diagnosis (*p* value of 0.82 and 0.67 respectively). Interestingly, Nikendei and co-authors found patients with metastasis had lower levels of psychological symptoms (*p* value 0.011) and higher levels of somatic symptoms (*p* value 0.014) when compared to non-metastatic cancer [8]. A greater sample size emphasizing the correlation between treatment of malignancy as well as depression on the overall outcome may be beneficial.

## Conclusion

Malignancy is associated with a greater risk of developing depressive disorders. The profile varies from other depressive disorder to MDD in different types of malignancies, and PHQ-9 serves as a good tool for screening and diagnosis, even in the patients presenting to ENT department with somatic as well psychological symptoms related to malignancy. Early detection of psychological conditions helps improve treatment adherence and performance score in malignancy patients.

## Electronic supplementary material

Below is the link to the electronic supplementary material.

Supplementary file1 (PDF 207 kb)

## Data Availability

Data transparent..
