# Supplementary material for: Depression profile in malignancy patients attending otorhinolaryngology clinic
Source: Eur Arch Otorhinolaryngol. 2020 Aug 17;278(2):537–41. doi: 10.1007/s00405-020-06289-w (PMC7826301; doi:10.1007/s00405-020-06289-w)
Supplement: Supplementary file 1 — Supplementary file1 (PDF 207 kb) [file 405_2020_6289_MOESM1_ESM.pdf]

PHQ-9 Questionnaire

**Over the last 2 weeks, how often have you been bothered by any of the following problems?** ***(Use "✓" to indicate your answer)***	**Not at all**	**Several days**	**More than half the days**	**Nearly everyday**
1. Little interest or pleasure in doing things	0	1	2	3
2. Feeling down, depressed, or hopeless	0	1	2	3
3. Trouble falling or staying asleep, or sleeping too much	0	1	2	3
4. Feeling tired or having little energy	0	1	2	3
5. Poor appetite or overeating	0	1	2	3
6. Feeling bad about yourself — or that you are a failure or have let yourself or your family down	0	1	2	3
7. Trouble concentrating on things, such as reading the newspaper or watching television	0	1	2	3
8. Moving or speaking so slowly that other people could have noticed? Or the opposite — being so fidgety or restless that you have been moving around a lot more than usual	0	1	2	3
9. Thoughts that you would be better off dead or of hurting yourself in some way	0	1	2	3

FOR OFFICE CODING: Total score = *0* + ______ + ______ + ______ =

If you checked off any problems, how difficult have these problems made it for you to do your work, take care of things at home, or get along with other people?

Not difficult at all	Somewhat difficult	Very difficult	Extremely difficult
☐	☐	☐	☐

From the Primary Care Evaluation of Mental Disorders Patient Health Questionnaire (PRIME-MD PHQ), developed by Drs. Robert L. Spitzer, Janet BW Williams, Kurt Kroenke, and colleagues. PRIME-MD is a trademark of Pfizer Inc. Copyright 1999 Pfizer Inc. May be photocopied *ad libitum*.
